# Supplementary material for: The HosA histone deacetylase regulates stress resistance, host cell interactions, and virulence in Aspergillus fumigatus
Source: Microbiol Spectr. 2026 Feb 19;14(4):e04036-25. doi: 10.1128/spectrum.04036-25 (PMC13055302; doi:10.1128/spectrum.04036-25)
Supplement: Fig. S1 — Stress susceptibility of the ΔhosA mutant. [file spectrum.04036-25-s0001.pdf]

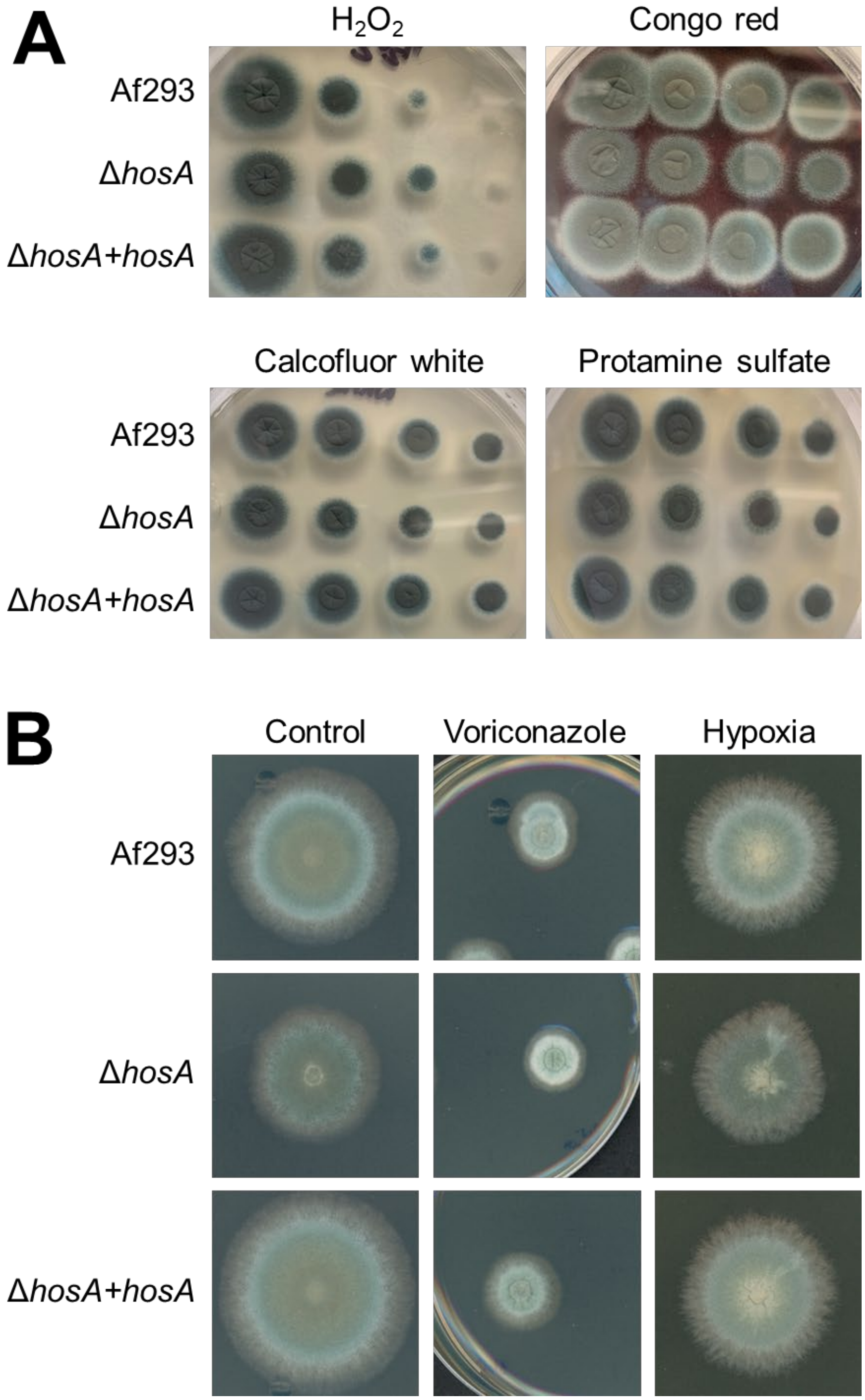

Fig. S1. Stress susceptibility of the Δ*hosA* mutant. (A) Susceptibility of the indicated *A. fumigatus* strains to 4 mM H_2O_2, 200 µg/ml Congo red, 300 µg/ml Calcofluor white, and 5 mM protamine sulfate. (B) Growth of the indicated strains in the presence of 0.25 µg/ml voriconazole or 1% O_2. Colonies were imaged after growth at 37°C for 48 h (A) or 60 h (B).
